# Extracellular lactate as an alternative energy source for retinal bipolar cells

**DOI:** 10.1016/j.jbc.2024.106794

**Published:** 2024-02-24

**Authors:** Victor Calbiague-Garcia, Yiyi Chen, Bárbara Cádiz, Felipe Tapia, François Paquet-Durand, Oliver Schmachtenberg

**Affiliations:** 1 PhD Program in Neuroscience, Universidad de Valparaíso, Valparaíso, Chile; 2 CINV, Instituto de Biología, Universidad de Valparaíso, Valparaíso, Chile; 3 Institute for Ophthalmic Research, University of Tübingen, Tübingen, Germany

**Keywords:** retina, bipolar cells, lactate, lactate shuttle, monocarboxylate transporters

## Abstract

Retinal bipolar and amacrine cells receive visual information from photoreceptors and participate in the first steps of image processing in the retina. Several studies have suggested the operation of aerobic glycolysis and a lactate shuttle system in the retina due to the high production of this metabolite under aerobic conditions. However, whether bipolar cells form part of this metabolic circuit remains unclear. Here, we show that the monocarboxylate transporter 2 is expressed and functional in inner retinal neurons. Additionally, we used genetically encoded FRET nanosensors to demonstrate the ability of inner retinal neurons to consume extracellular lactate as an alternative to glucose. In rod bipolar cells, lactate consumption allowed cells to maintain the homeostasis of ions and electrical responses. We also found that lactate synthesis and transporter inhibition caused functional alterations and an increased rate of cell death. Overall, our data shed light on a notable but still poorly understood aspect of retinal metabolism.

The retina is a neural tissue in which visual signals are transduced by photoreceptors and subsequently processed by inner retinal neurons. The constant turnover of the photopigment in the outer segments and the large ATP consumption required to keep the ion pumps working to maintain the dark current in the inner segment of photoreceptors are among the main reasons why the retina is the most energy-demanding neural tissue (1, 2, 3, 4). Photoreceptors receive glucose, oxygen, and other metabolites from the choroidal vasculature (5) through the retinal pigment epithelium (RPE (6)). However, the inner retina may either be vascular or avascular. In avascular retina (*e.g.*, guinea pig and rabbit), bipolar cells (BCs) and amacrine cells (ACs) probably rely only on Müller cells (MCs) as energy providers, while in vascular retinas (*e.g.*, rats, mice, and primates), these neurons can take up fuel directly from the inner retinal capillaries, apart from MCs and astrocytes (5, 7).

Interestingly, Otto Warburg's data from 1924 already suggested that the retina mostly utilizes aerobic glycolysis to generate ATP, converting almost 70% of the consumed glucose into lactate (8, 9), and many later studies have supported the idea of a retinal lactate shuttle (10, 11, 12). Although the metabolic conditions and the site of lactate release remain unclear, these investigations support the notion of a “metabolic ecosystem” between MCs, RPE, and photoreceptors, in which lactate produced by one cell type may be consumed by others. However, the dynamics and the extension of this shuttle into the inner retina remain a matter of debate, especially in the vascular retina (7, 13). Prior studies have shown that inhibiting monocarboxylate transporters (MCTs) (14), and deleting basigin (15), an accessory protein linked to monocarboxylate transporter one (MCT1), result in a reduction of the electroretinogram (ERG) b-wave amplitude and of the oscillatory potentials. Because the ERG b-wave is generated by ON BCs, these reports support the importance of lactate for electrical signaling in the inner retina. This alteration could be partially ameliorated by exogenous lactate, revealing a potential for lactate uptake by inner retinal neurons (14). This idea is supported by other studies in mice, where the genetic deletion of aerobic glycolysis markers such as pyruvate kinase M2 (PKM2) (expressed in photoreceptors), and lactate dehydrogenase A (LDH-A) (expressed in photoreceptors and MCs), decreased the ERG a-wave (16) and b wave (16, 17), suggesting that lactate produced by photoreceptors is consumed in the inner retina and is needed to maintain the ERG response.

Taking these antecedents into account, we hypothesize a consumption of lactate by inner retinal neurons to support and maintain their physiological activity. Yet, single-cell studies to determine which substrate each cell type consumes under physiological conditions are lacking.

Thus, we set out to test the role of extracellular lactate as a putative alternative energy substrate for mouse retinal BCs. To this end, we expressed a genetically-encoded FRET-based lactate sensor. These types of sensors are fusion proteins composed of a ligand-binding moiety, recognition element, and fluorescent pair that allowed us to qualitatively determine the levels of some metabolites in real time (18, 19). The possible role of lactate as a substrate for the physiological activity of inner retinal neurons was tested by calcium imaging and electrophysiological recordings. These measurements were complemented by markers of specific enzymes involved in aerobic glycolysis and the pharmacological inhibition of lactate transporters and enzymes related to aerobic glycolysis.

## Results

### The inner retina expresses MCTs

**Figure 1:**
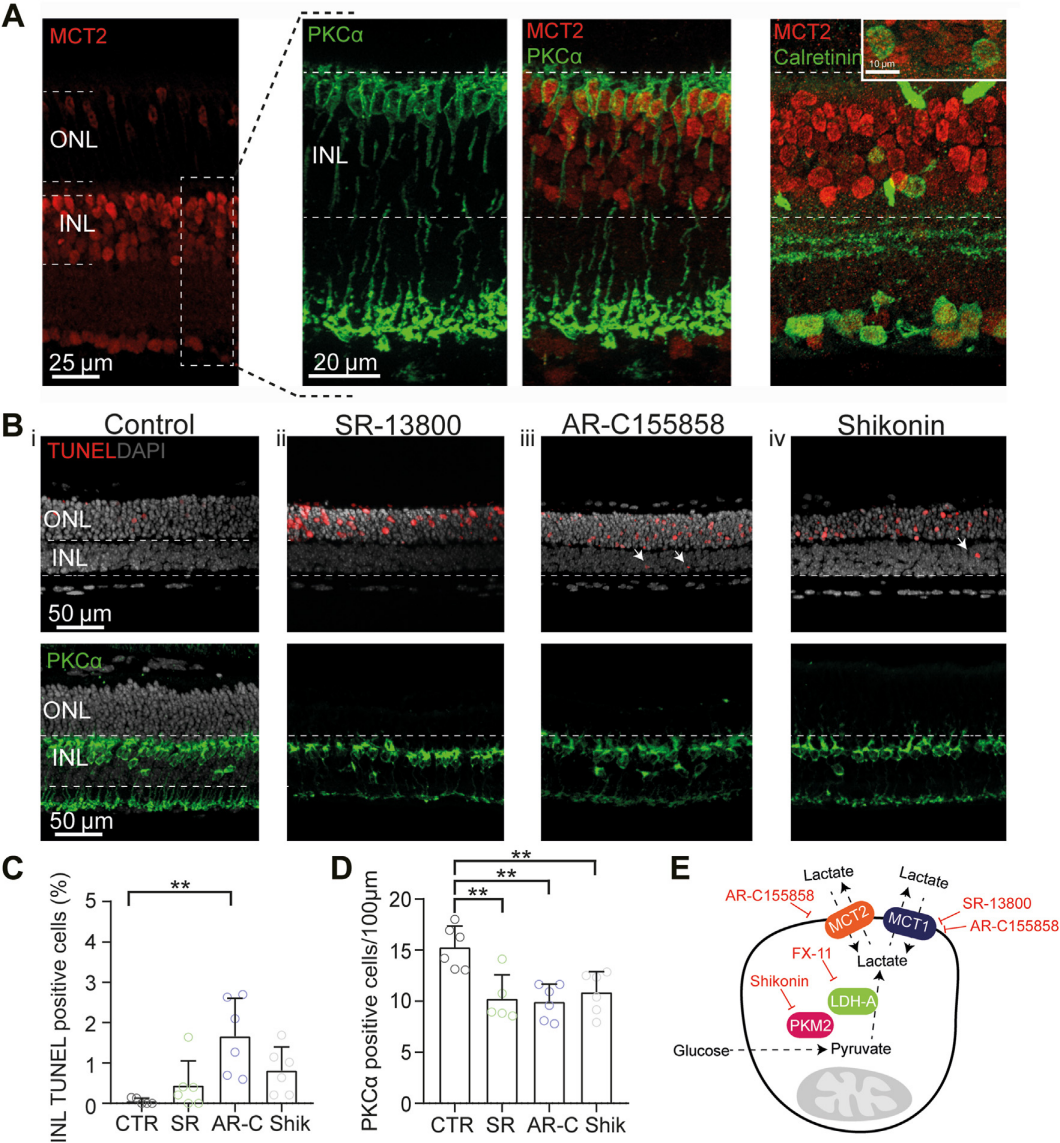
**Lactate metabolism is required for inner retinal neuron survival.***A*, immunofluorescence labeling of retinal transverse cryosections showing MCT2 expression mainly in the inner nuclear layer (INL). Colabeling for PKCα and calretinin illustrates MCT2 expression in RBCs and a subset of ACs. *B*_i_–*B*_iv_ (*top*), (*C*) statistical analysis, and quantification of the cell death assay (TUNEL), performed in organotypic retinal explants, showing occasional TUNEL positive nuclei (*arrows*). When compared to control (CTR, n = 5) treatment with AR-C and Shikonin increased INL cell death, while SR treatment did not. The data were analyzed with the Kruskal-Wallis and Dunn’s multiple comparison post hoc tests. B_i_-B_iv_ (*bottom*), (*D*) quantification of RBCs per 100 μm retinal length revealed a significant density reduction of this cell type after treatment with SR, AR-C, and Shikonin, supporting a dependence of RBCs on extracellular lactate. The data were analyzed by one-way ANOVA with Tukey’s multiple comparison post hoc test. Each *dot* reflects a single retinal explant. *E*, schematic summary, showing the transporters, applied drugs used throughout the investigation, and their respective effects on lactate metabolism. Shikonin and FX-11 inhibit lactate synthesis directly, while AR-C155858 and SR-13800 block lactate transport. Graphs display mean values ± SD; *asterisks* indicate ∗*p* < 0.05, ∗∗*p* < 0.01. SR = MCT1 inhibitor; Shikonin = PKM2 inhibitor. ACs, amacrine cells; AR-C = MCT2 inhibitor; INL, inner nuclear layer; LDH-A, lactate dehydrogenase A; MCT1, monocarboxylate transporter one; MCT2, monocarboxylate transporter 2; ONL, outer nuclear layer; PKCα, protein kinase Cα; PKM2, pyruvate kinase M2; RBCs, rod bipolar cells.

To elucidate the potential role of extracellular lactate in the inner retina, we examined the expression pattern of MCT2 in the retina of WT mice. MCTs are proton-linked plasma membrane transporters that allow the transport of lactate and pyruvate into and out of cells (20). We focused on the MCT2 isoform because it has been reported to be the prototypical neuronal importer of lactate (21), especially in high-lactate environments (22). Immunofluorescence localization indicated that MCT2 was expressed in a subset of somas in the outer nuclear layer, likely cones, but is more strongly and abundantly found in cell bodies of the inner nuclear layer (INL, Fig. 1*A*). We performed coimmunostaining with different cell markers to identify inner retinal neurons expressing MCT2. For this purpose, we used protein kinase Cα to label rod bipolar cells (RBCs), and calretinin to label ACs. These coimmunostainings indicated that MCT2 was expressed in both cells types in the INL (Fig. 1*A*).

### Inhibition of lactate transport increased cell death in the inner retina

The widespread expression of MCT2 in the INL led us to hypothesize that extracellular lactate may be used by inner retinal cells to meet their physiological demands. To study the role of extracellular lactate in the inner retina as a first functional approach, we cultured mouse organotypic retinal explants for 7 days to allow us to perform experiments under fully controlled *in vitro* conditions (23). Since there are no commercial inhibitors selective for only MCT2, we treated retinal explants with MCT1 or MCT1/MCT2 inhibitors, to separate the roles of MCT1 and MCT2: SR-13800 (24) for MCT1, and AR-C155858 for MCT1/MCT2 (25).

To confirm the effects of these inhibitors in retinal lactate production and release, we first measured the lactate concentration in the medium of retinal explant cultures on consecutive days. Under inhibition of MCT1 with SR-13800, retinal explants showed an extracellular lactate concentration of 12.8 ± 0.8 mM (n = 3), indicating a decrease of lactate release into the culture medium after 4 days of treatment compared to untreated controls (16.6 ± 1.1 mM, n = 3). On the other hand, after addition of AR-C155858, the lactate concentration in the medium of retinal explants was 20.3 ± 1.1 mM (n = 3), indicating an accumulation of lactate after 4 days (Fig. S1).

We then used the TUNEL assay to examine whether MCT2 function was essential for cell survival in culture, quantifying the cell death rate as the fraction of apoptotic cells in the INL after 4 days of treatment. In the control condition, the cell death rate was 0.06 ± 0.07% (n = 5), and a comparatively low rate (0.44 ± 0.61%, n = 6, *p* = 0.63) was found in the INL (SR, Fig. 1, *B* and *C*) under SR-13800 treatment. On the other hand, addition of AR-C produced a significant increase (1.67 ± 0.94%, n = 6, *p* = 0.0014) of TUNEL-positive cells (Fig. 1, *B* and *C*), indicating that MCT2 function was relevant for the survival rate of INL cells.

To test the consequences of a reduction of retinal lactate synthesis, we inhibited the enzyme PKM2 with Shikonin (26). Pyruvate kinase is a key glycolytic enzyme that controls the final step of glycolysis, converting phosphoenolpyruvate to pyruvate, and the M2 isoform is a key regulator of aerobic glycolysis specifically associated with tumor cells (27). As with the MCT blockers, we first tested the overall effect of this drug on the lactate concentration in the medium of retinal explant cultures. Although the inhibition of PKM2 by Shikonin did not produce a decrease of lactate levels compared to control explant cultures (Fig. S1), the retinal explants treated with Shikonin displayed an average progressive decrease of 3.2 ± 2.1 mM of lactate after 4 days of culture (n = 3, Fig. S1). In the TUNEL assay, the inhibition of PKM2 resulted in a small increase in INL-specific cell death (0.82 ± 0.57%, n = 6, *p* = 0.05) (Fig. 1, *B* and *C*). Notably, under all tested conditions, the number of TUNEL-positive cells in the outer nuclear layer was much higher: SR = 19.9 ± 6.9%, n = 6, *p* < 0.0001, AR-C = 16.6 ± 5.2%, n = 6, *p* = 0.0007, Shikonin = 14.5 ± 5.9%, n = 6, *p* = 0.002, reflecting the sensitivity of photoreceptors to metabolic disruptions (6).

After confirmation of MCT2 expression in RBCs, we set out to explore the effects of the inhibition of lactate transport and production on the survival rate of this specific cell type. To this end, the number of RBCs per 100 μm retinal section length was counted, yielding 15.3 ± 2.1 cells (n= 5, Fig. 1, *B* and *D*) in untreated control explants. Interestingly, in all treatments, the RBC number decreased compared to the control condition: SR (10.2 ± 2.3, n = 6, *p* = 0.003), AR-C (9.9 ± 1.7, n = 6, *p* = 0.001), and Shikonin (10.8 ± 2.0, n = 6, *p* = 0.007), suggesting that lactate production and transport were important for maintaining RBCs alive. Taken together, these results indicate that inner retinal cell survival is sensitive to the inhibition of lactate metabolism, and specifically, that RBCs need lactate for survival in culture.

### Lactate transport is important to maintain neuronal depolarization in the inner retina

**Figure 2:**
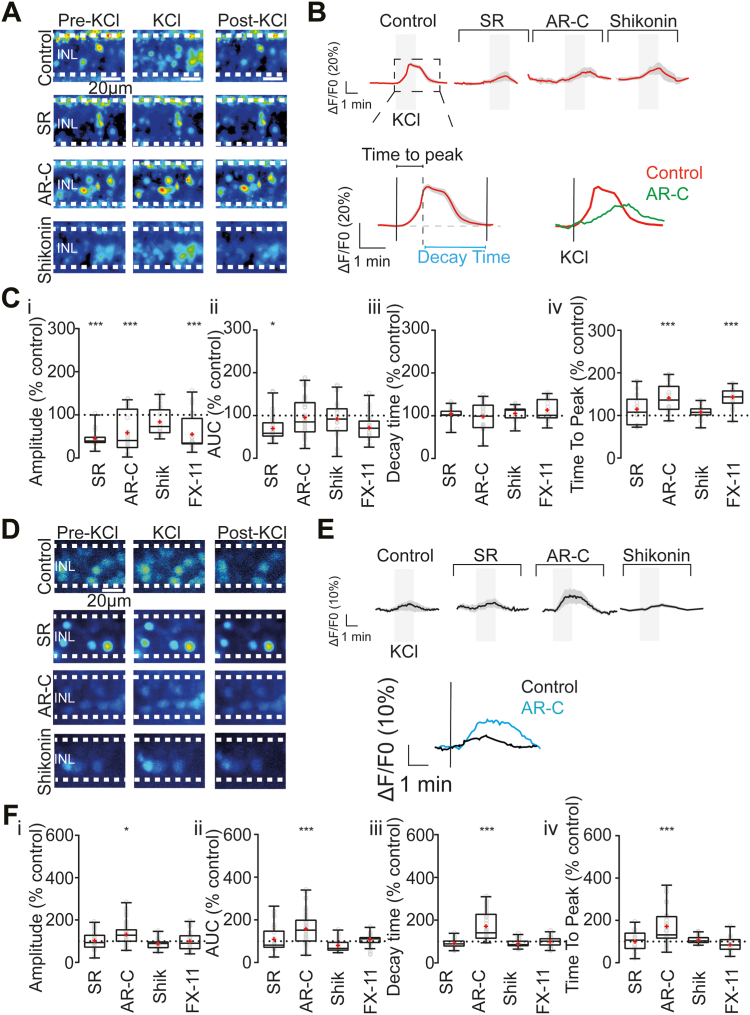
**Depolarization of inner retinal cells is reduced and slowed by inhibition of lactate metabolism.***A* and *D*, representative images of Fluo4-AM and CoroNa Green-loaded cells in the INL, before and at two time points after bath perfusion with KCl, in controls and retinas incubated with SR, AR-C, and Shikonin. *B* and *E*, (*top*), traces of individual experiments in each condition (*red line* = mean, *gray shadow* = SD). *B* and *E*, (*bottom*), overview of the kinetic parameters measured in the imaging. *C*_i–iv_, statistical analysis of the different parameters in the calcium imaging experiments indicating alterations in the amplitude and time to peak. *F*_i–iv_, statistical analysis of the sodium imaging experiments showing specific alterations in all the parameters after MCT2 inhibition. Box plots display the median ± min and max values and the mean in *red*. Individual values are displayed as *open circles* (*gray*). The control is represented as a *dashed line* at 100%, and results are presented as percentage of control. *Asterisks* indicate ∗ *p* < 0.05, ∗∗*p* < 0.01, ∗∗∗*p*< 0.001. INL, inner nuclear layer; MCT, SR= MCT1 inhibitor; AR-C= MCT2 inhibitor; Shikonin = PKM2 inhibitor; FX-11= LDH-A inhibitor. See also Figs. S1 and S2. LDH-A, lactate dehydrogenase A; MCT, monocarboxylate transporter.

Given that the principal function of neurons is propagating electrical signals, it can be assumed that this activity depends to some degree on their energy metabolism. Yet, whether inner retinal cells use lactate apart from glucose to fuel their physiological activity remains unclear. To address this question, we performed calcium imaging experiments, which allows for a global assessment of retinal function. To this end, acute retinal slices from WT P30 mice were incubated for 15 min with two MCT inhibitors (Fig. 2, *A* and *B*), and the change in the relative fluorescence intensities of BC and AC cell bodies in the INL was measured as the amplitude and area under the fluorescence intensity curve (AUC) of the responses after a slight depolarization through K^+^ stimulation under photopic conditions (Fig. 2*B*). For this and the following results, the data were normalized *versus* control conditions (dashed line at 100%), and the *p*-value indicates significance compared to control. Control conditions correspond to acute retinal slices from WT P30 mice incubated with vehicle (dimethyl sulfoxide). Under blockage of MCT1 (n = 3 retinas, region of interests (ROIs) = 24) and MCT1/MCT2 (n = 3 retinas, ROIs = 30), the calcium signal amplitudes were reduced to 44.5 ± 19.0% (*p* = 0.003) and 58.5 ± 45.0% (*p* = 0.0004) of control (Fig. 2, *B* and *C*), respectively. Interestingly, acute inhibition of PKM2 with Shikonin (n = 3, ROIs = 20) had no noticeable impact on the amplitude of the calcium responses (Fig. 2, *B* and *C*, 84.1 ± 32.4%, *p* = 0.999), suggesting that Shikonin application may become effective in reducing the lactate release only after prolonged treatment (*e.g.*, incubation of retinal explants), supporting the notion that inner retinal cells depend on lactate uptake rather than its production through glycolysis.

Subsequently, we set out to test the role of lactate production in the retina through inhibition of the key enzyme LDH-A with the drug FX-11 (28). This enzyme is directly responsible for the conversion of pyruvate to lactate. As with PKM2, it is a fundamental regulator of aerobic glycolysis and is frequently overexpressed in tumor tissue (29). Inhibition of LDH-A should decrease the aerobic glycolysis rate, and thereby lactate levels (Fig. S1). Under this condition (n = 3, ROIs = 23), the response amplitude was again reduced compared to controls (Fig. 2*C*; 55.1 ± 39.9%, *p* = 0.0001). In contrast, the AUC did not change in the conditions of MCT blockage with either AR-C (Fig. 2*C*, 95.6 ± 43.1%, *p* = 0.995), Shikonin (Fig. 2*C*, 92.2 ± 43.6%, *p* = 0.968), or FX-11 (Fig. 2*C*, 71.9 ± 30.4%, *p* = 0.205), and was only affected in the SR condition (Fig. 2*C*, 69.9 ± 30.2%, *p* = 0.015), suggesting that the response kinetics were differentially affected by the drugs. To further analyze this hypothesis, we measured the time-to-peak and the decay time of the responses. We observed an increase in the time-to-peak when MCT1/MCT2 were inhibited (Fig. 2, *B* and *C*, 140.9 ± 31.7%, *p* < 0.0001) and under FX-11 treatment (Fig. 2*C*, 143.2 ± 23.8%, *p* < 0.0001), while MCT1 inhibition (114.9 ± 34.5%, *p* = 0.251) and PKM2 inhibition (108.0 ± 15.8%, *p* = 0.514) had no effect. However, the decay time was unaffected in all conditions (Fig. 4*C*): SR (105.0 ± 13.8%, *p* = 0.724), AR-C (98.2 ± 30.8%, *p* = 0.995), Shikonin (105.6 ± 15.6%, *p* = 0.999), and FX-11 (113.4 ± 25.5%, *p* = 0.732). Altogether, these results indicate that INL cells are susceptible to inhibition of lactate synthesis and transport causing a disruption of their calcium responses to transient depolarization.

Since our slight depolarization through K^+^ stimulation was applied to all retinal neurons, we also measured the amplitude and AUC of photoreceptor calcium responses to clarify if a disruption in photoreceptor activity may cause the observations in INL cells. Interestingly, photoreceptors only showed a reduction in calcium signal amplitudes when MCT1 (58.1 ± 32.9%, n = 3, ROIs = 58, *p* = 0.0004) and PKM2 (63.9 ± 36.1%, n = 3, ROIs = 70, *p* = 0.0032) were blocked (Fig. S1). Under blockage of MCT2 (70.6 ± 40.9%, n = 3, ROIs = 60, *p* = 0.0846) and inhibition of LDH-A (111.9 ± 90.5%, n = 3, ROIs = 78, *p* > 0.9999), no effects were observed (Fig. S1). Regarding AUC, no changes were observed in any of the conditions (Fig. S1). These results reveal that the changes in calcium responses of INL cells after blockage of MCT2 and inhibition of LDH-A are not due to a side effect of photoreceptor alterations. In fact, only the effect of MCT1 blockage may be influenced by photoreceptors. The unaffected response in decay time in INL cells was initially surprising because a disruption of energy consumption should affect the restoration of ion gradients after depolarization (30). However, because the calcium concentration is critical for cells, they have redundant intracellular buffers, which can quickly restore cytosolic calcium levels (31). Therefore, the next step was to directly evaluate ion pump function. We used the sodium probe CoroNa Green (Fig. 2, *D* and *E*) to assay the importance of lactate on sodium flux. Neither inhibition of MCT1 (n = 3, ROIs = 33, 102.1 ± 40.9%, *p* > 0.999), PKM2 (n = 3, ROIs = 20, 87.6 ± 26.1%, *p* > 0.999), nor inhibition of LDH-A (n = 3, ROIs = 29, 100.0 ± 45.7%, *p* > 0.999) caused significant effects (Fig. 2*F*). Similar results were observed for the AUC: SR (108.9 ± 64.2%, *p* > 0.999), Shikonin (80.7 ± 30.9%, *p* = 0.318), and FX-11 (108.3 ± 23.1%, *p* = 0.965). However, when we inhibited MCT1/MCT2 with AR-C, the amplitude (131.8 ± 51.5%, *p* = 0.007) and AUC (157.1 ± 70.9%, *p* = 0.0002) of the sodium responses increased significantly (Fig. 2*F*).

Similar results were obtained regarding the other response kinetic parameters: neither MCT1 (100.3 ± 41.7%, *p* > 0.999), PKM2 (107.2 ± 19.4%, *p* > 0.999), nor LDH-A (85.9 ± 31.7%, *p* = 0.424) inhibition caused changes in the time-to-peak. When we analyzed the decay time, no changes were observed either: MCT1 (90.9 ± 21.5%, *p* = 0.823), PKM2 (89.7 ± 18.9%, *p* = 0.664), and LDH-A (99.9 ± 26.3%, *p* > 0.999). But inhibition of both MCT1/2 with AR-C significantly affected the time-to-peak (171.0 ± 85.4%, *p* < 0.0001), and increased the response decay time (171.0 ± 70.2%, *p* < 0.0001) (Fig. 2*F*).

Taken together, these results indicate that MCT2 inhibition was necessary and sufficient to affect retinal response kinetics to depolarization, suggesting an alteration in ion pumping to restore sodium homeostasis (30, 32). If this idea is indeed correct, changes in the basal levels of calcium and sodium should also be observable. Therefore, we measured basal fluorescence levels and calculated the slope of the relative fluorescence to analyze whether calcium or sodium accumulation occurred under inhibition of the different transporters and enzymes related to aerobic glycolysis.

As for the relative calcium levels, it was striking to see that under three different conditions (*i.e.*, SR, Shikonin, and FX-11), they decreased significantly: Blockage of MCT1 caused a reduction of 74.8 ± 43.9% (*p* < 0.0001), PKM2 of 77.1 ± 85.6% (*p* < 0.0001) and LDH-A of 147.1 ± 78.0% (*p* < 0.0001) (Fig. S2). In contrast, incubation with AR-C resulted in a slight increase in relative intracellular calcium levels (34.9 ± 79.2%, *p* = 0.015; Fig. S2, *B* and *E*).

Conversely, for relative sodium levels, only the inhibition of MCT2 and, to a lesser extent, of LDH-A had an effect (Fig. S3). A significant drop in the relative sodium level of 282.2 ± 155.1% (*p* < 0.0001) was seen under AR-C incubation, while FX-11 caused a minor reduction of 31.9 ± 55.7% (*p* = 0.001) (Fig. S3). In summary, these results demonstrate that inhibition of lactate metabolism can disrupt several features of neuronal depolarization in inner retinal cells, and support the idea of a specific expression of MCT2 over other aerobic glycolysis enzymes in the inner retina.

### Lactate can sustain different RBC currents

To collect further evidence for the involvement of lactate in inner retinal neuron physiology, we set out to measure representative voltage-gated currents in retinal cells performing whole-cell patch-clamp recordings under different lactate and glucose concentrations. In mammals, the concentration of lactate in the retina has been reported to fluctuate between 5 and 50 mM, depending on the species (33). Here, we measured the lactate released by retinal explants after 7 days in culture and obtained a concentration of 16.6 ± 1.1 mM lactate in the culture medium, in line with previously reported values (33) (Fig. S1).

Then, acutely isolated retinas from P30 mice were incubated for 15 min in four different conditions under photopic conditions: 20 mM glucose (control), 20 mM lactate + 10 mM mannitol (no glucose), 40 mM lactate (no glucose), and 20 mM mannitol (no glucose; mannitol was used for osmolarity compensation). Subsequently, the outward currents (*I*_*out*_), the calcium current (*I*_*Ca*_) and the membrane potential (*V*_*memb*_) were measured under photopic conditions.

These electrophysiological experiments showed that in retinas incubated with 20 or 40 mM lactate, RBCs did not change the amplitudes of their *I*_*Ca*_, *I*_*out*_, and *V*_*memb*_ compared to retinas incubated with glucose (Fig. S4 and Table 1). Retinal cells incubated with 20 mM mannitol only showed a decrease in *I*_*Ca*_ and *I*_*out*_ and an increase in *V*_*memb*_ (Fig. S4 and Table 1). A specific feature of the calcium current in RBCs is the presence of transient reciprocal feedback from A17 cells (34). This feedback was only affected in the presence of 40 mM lactate and was abolished under mannitol conditions (Fig. S4 and Table 1). In contrast, the 20 mM lactate condition maintained the amplitude of this reciprocal feedback current. These results indicate that 20 mM lactate can be a functional substitute for glucose, suggesting that RBCs can use this metabolite as an alternative substrate to maintain their physiological activity. Moreover, a saturated lactate environment and glucose deprivation conditions affect A17 feedback.

**Table 1:** Summary of the principal electrophysiological characteristics of RBCs under different metabolic conditions

Condition	*I*_*out*_ out (pA)	*I*_*Ca*_ (pA)	Reciprocal feedback (pA)	*V*_*memb*_ (mV)
Glucose	775.9 ± 73.2 (12)	20.8 ± 3.7 (7)	26.5 ± 10.4 (4)	−41.2 ± 5.4 (10)
Lactate 20 mM	729.2 ± 140.3 (9)	19.0 ± 2.8 (8)	23.1 ± 4.5 (6)	−41.1 ± 7.6 (9)
Lactate 40 mM	841.7 ± 96.7 (8)	17.2 ± 2.1 (6)	3.6 ± 1.7 (4)	−40.8 ± 6.5 (7)
Mannitol	364.6 ± 86.7 (9)	8.2 ± 4.3 (4)	--- (4)	−31.5 ± 6.5 (7)
Gluc + SR	667.8 ± 147.9 (8)	18.9 ± 5.5 (3)	28.3 ± 9.9 (3)	−36.1 ± 7.4 (8)
Gluc + AR-C	677.5 ± 84.3 (8)	21.8 ± 6.7 (3)	28.5 ± 2.1 (3)	−37.6 ± 5.3 (9)
Lact 20 mM + SR	633.8 ± 173.3 (8)	20.6 ± 6.7 (5)	28.5± 5.4 (5)	−35.8 ± 5.7 (8)
Lact 20 mM + AR-C	438.0 ± 95.9 (5)	8.4 ± 3.8 (8)	11.3 ± 4.8 (5)	−31.2 ± 4.9 (5)

Values are given as mean ± SD, and total number of cells (n).

**Figure 3:**
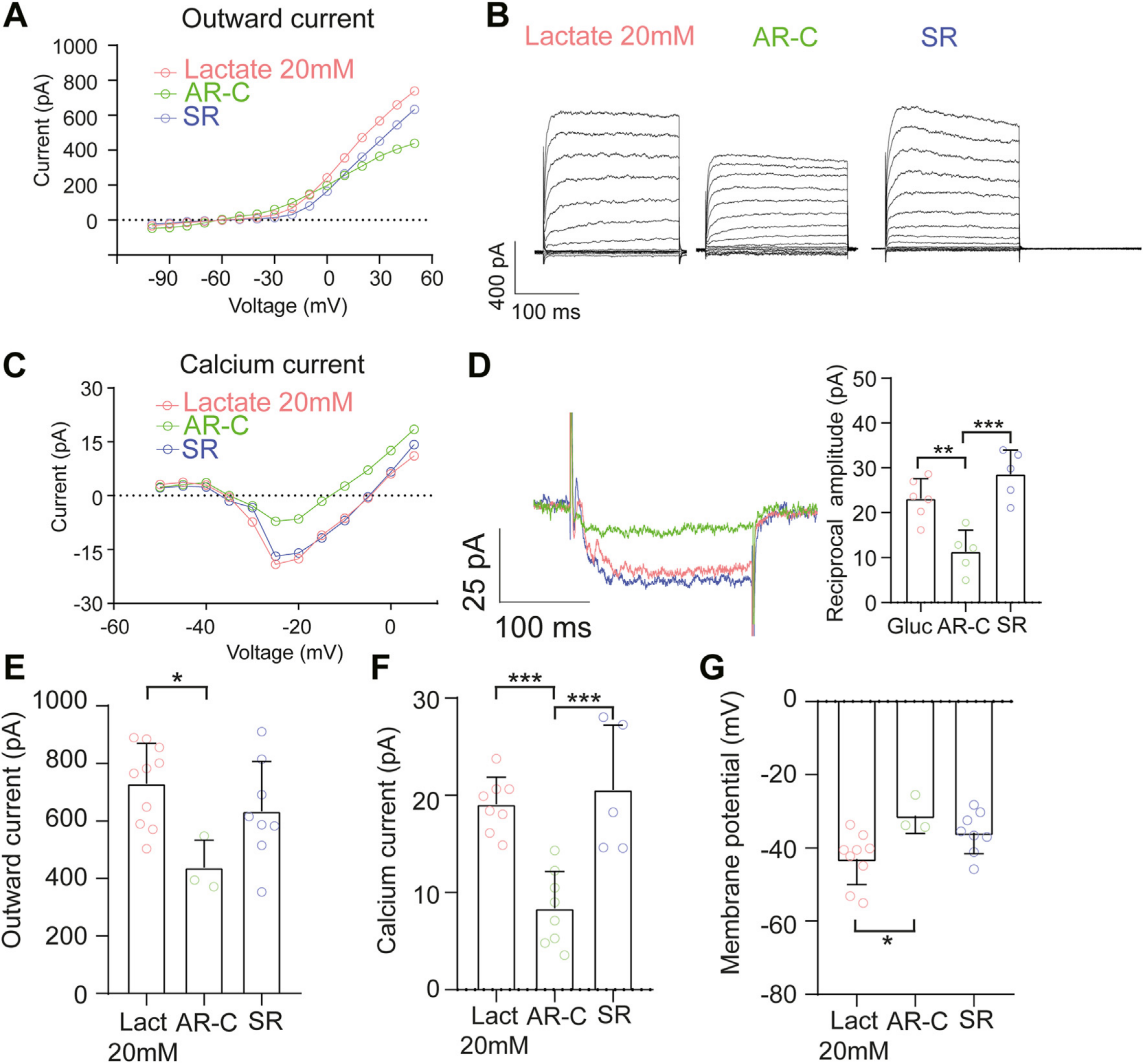
**Inhibition of lactate transport through MCT2 induces alterations in RBC currents.***A* and *C*, comparison of the voltage-current relationship of the outward current and calcium current under different conditions. *B* and *D*, representative recordings to depolarizing voltage steps. Reciprocal feedback was altered only in the AR-C condition (*p* = 0.0042) but was unaffected in the SR condition (*p* = 0.1957) compared to controls. *E* and *F*, in the presence of 20 mM lactate, we observed a decrease in the outward current (*p* = 0.0224) and calcium current (*p* = 0.0003) only under MCT2 inhibition, but no differences were noted either in the outward current (*p* = 0.3925) or calcium current (*p* = 0.8146) when only MCT1 was blocked. *G*, similar results were obtained when we measured the membrane potential, where AR-C (*p* = 0.0321) caused a significant depolarization, while the SR condition was not different from controls (*p* = 0.0822). Data were analyzed using one-way ANOVA, followed by Tukey’s multiple comparison post hoc test. Each *dot* represents a single recorded cell. Graphs represent the mean ± SD; *asterisks* indicate ∗ *p* < 0.05, ∗∗*p* < 0.01, ∗∗∗*p*< 0.001. SR = MCT1 inhibitor; AR-C= MCT1 and MCT2 inhibitor. See also Fig. S3 and S4, and Table 1. MCT, monocarboxylate transporter; RBC, rod bipolar cell.

To test functional MCT2 expression in RBCs, retinal slices were incubated with different MCT inhibitors in the 20 mM lactate condition, because this concentration triggers an influx of this metabolite (see below). When we measured the above parameters, we observed that alterations occurred only when MCT1 and MCT2 were inhibited simultaneously, but not when only MCT1 was inhibited (Fig. 3 and Table 1). These results are in line with the immunolabeling and imaging experiments, supporting MCT2 expression in RBCs and its relevance for neuronal activity.

Finally, we wondered how lactate compared to glucose as a fuel suitable to meet the physiological demands of RBCs. To this end, we applied 20 mM glucose and blocked the different MCTs. As expected, neither AR-C nor SR altered the amplitude of *I*_*out*_, *I*_*Ca*_, *V*_*memb*_, or reciprocal feedback currents (Fig. S5 and Table 1), suggesting that RBCs can use both glucose and lactate alternatively to sustain their physiology.

### Intracellular lactate dynamics in inner retinal cells

Previous results have supported the idea of lactate consumption in inner retinal cells (14), nonetheless, intercellular and intracellular lactate flux in the inner retina remain obscure. To investigate lactate dynamics in INL cells, we expressed the FRET nanosensor h-Syn-Laconic (neurons) and glial fibrillary acidic protein (GFAP)-Laconic (MCs) in organotypic retinal explants under photopic conditions, as previously described (18, 35). First, we evaluated whether nanosensor expression was functional in inner retinal neurons, by measuring the ratio between the emissions obtained from monomeric teal fluorescent protein (mTFP)/Venus in the soma of putative BCs and ACs in the INL (Fig. S6). Subsequently, 10 mM lactate was added to the extracellular solution (Fig. S6), since at this concentration the nanosensor becomes saturated (35). To deplete the intracellular levels of these metabolites, we used 10 mM pyruvate to exploit the trans-acceleration property of MCTs (36). The delta ratio between depletion and saturation for Laconic was 20.3 ± 4.1% (Fig. S6), which is in line with the responses of the retina and other cells and tissues (18, 35). Together, these results showed that FRET nanosensors can be functionally expressed in retinal explant cultures to study neuronal metabolic dynamics on a single-cell level.

**Figure 4:**
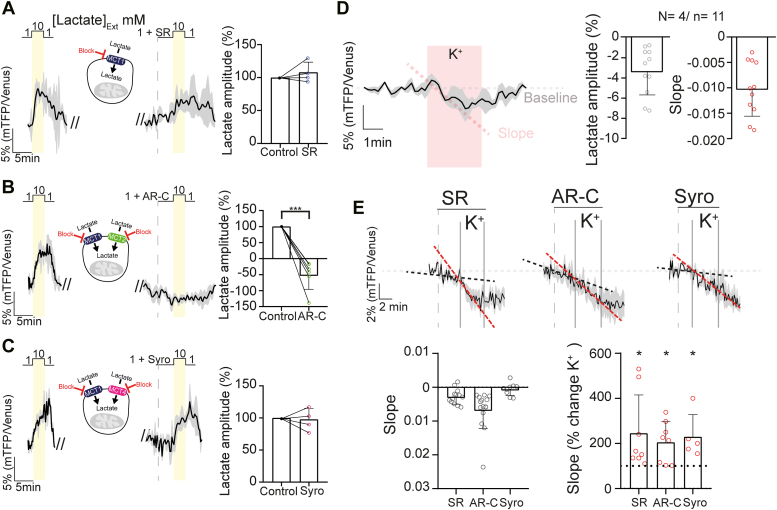
**Combined MCT1/MCT2 inhibition decreases intracellular lactate levels in inner retinal neurons.***A*–*C*, (*left*), from *left* to *right*: fluorescence curves showing lactate influx evoked by stimulation with 10 mM lactate, a diagram showing the transporters affected by the application of each drug and a fluorescence trace showing the effect of each drug on the lactate influx. *A*–*C*, (*right*), statistical analysis of the response amplitude. An evident response disruption was noted only when MCT2 was inhibited. However, under MCT1 and MCT4 inhibition, the amplitude was unaltered. *D*, modulation of intracellular lactate levels by transient depolarization. *E*, (*top*), effects of the inhibition of different MCTs and retinal depolarization on lactate dynamics. *Black dashed lines* indicate drug-induced change in slope and *red dashed lines* indicate the effect of drug + KCl. *E*, (*bottom*), statistical analysis of the slope of the responses under basal conditions (only drug, *left*) and depolarization (drug + K^+^, *right*). The data revealed that MCT2 inhibition led to a reduction in intracellular lactate levels, resulting from lactate consumption. This consumption was exacerbated after depolarization in SR, AR-C, and Syro. Data were analyzed using either a paired Student’s *t* test or Wilcoxon matched-pair test. The *black trace* represents the average of one experiment, whereas the *light gray shadow* represents the SD. Graphs display the mean ± SD. ∗ Indicates *p* < 0.05. SR = MCT1 inhibitor; AR-C = MCT1 and MCT2 inhibitor; Syro = MCT1 and MCT4 inhibitor. The number of experiments is represented as N = number of explants and n = number of cells recorded. See also Figs. S5 and S6. MCT, monocarboxylate transporter.

After confirming the functional expression of the Laconic lactate sensor in inner retinal neurons, we aimed to verify the function of MCT2 in the INL. Since these transporters work bidirectionally, we applied 10 mM lactate stimulation to trigger lactate influx into cells, which produced a fluorescence increase of 7.9 ± 3.4% (Fig. 4). Under inhibition of MCT1 (SR = 9.9 ± 3.6%, *p* = 0.377) or MCT1 and MCT4 with Syrosingopine (Syro = 9.0 ± 3.1%, *p* = 0.819), this fluorescence increase was unaffected (Fig. 4). However, when MCT1 and MCT2 were blocked, lactate influx was abolished (Fig. 4*B*, AR-C = −1.9 ± 0.6%, *p* = 0.0005), confirming the predominant role of MCT2 for lactate flux in inner retinal neurons. Overall, these results confirm the functional expression of MCT2 in BCs and ACs.

We then investigated how lactate dynamics change under different conditions in retinal neurons. To that end, we compared the responses under basal conditions and under moderate depolarization with 12 mM KCl. A moderate depolarization triggered a transient decrease in intracellular lactate, as evidenced by a −4.6 ± 2.0% fluorescence drop in inner retinal neurons, suggesting lactate consumption (Fig. 4*D*). However, it is important to note that one-third of the cells recorded (35.3%) did not respond to this stimulation (Fig. S7).

**Figure 5:**
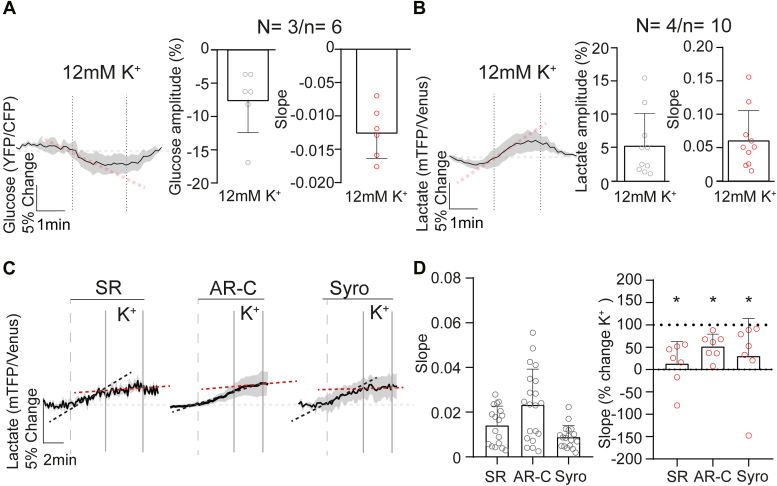
**Lactate dynamics of MCs under different conditions.***A* and *B*, effect of transient depolarization on intracellular glucose (*A*) and lactate (*B*) levels. *C*, lactate dynamics after inhibition of different MCTs and retinal depolarization. *Black dashed lines* indicate drug-induced changes in slope and *red dashed lines* indicate the effect of drugs + KCl. *D*, statistical analysis of the slope of the responses in the basal condition (drug only) and under depolarization (drug + K+). Accumulation of intracellular lactate was observed after bath application of different MCT inhibitors. However, this increase was less intense after depolarization under all conditions. Data were analyzed either with paired Student’s *t* test or Wilcoxon matched-pairs test. Graphs display the mean ± SD. ∗ Indicates *p* < 0.05. SR = MCT1 inhibitor; AR-C = MCT1 and MCT2 inhibitor; Syro = MCT1 and MCT4 inhibitor. The *black trace* represents the average response, while the *light gray shadow* represents the SD. The number of experiments is represented as: N = number of explants; n = number of cells recorded. MCs, Müller cells; MCT, monocarboxylate transporter.

To compare the lactate dynamics in different inner retinal cells, we performed the same experiment in MCs, to separate neuronal and nonneuronal components. In these cells, depolarization produced an increase in lactate levels (4.9 ± 4.1%; Fig. 5*B*). We also expressed a glucose nanosensor (Δ6) to analyze the glucose levels in MCs. Remarkably, depolarization triggered a transient decrease in MCs intracellular glucose, as evidenced by a fluorescence drop measured using the Δ6 nanosensor (−7.5 ± 4.9%; Fig. 5*A*), suggesting higher glucose consumption triggered by K^+^ in MCs. Interestingly, this result is in line with data obtained from astrocytes in the brain (37).

Overall, these results revealed a potential difference in metabolism between neurons and MCs in the retina, where neurons may consume lactate, whereas MCs may produce it. To test this hypothesis, we performed a transport-stop protocol (18, 38, 39). After inhibition of MCTs, the intracellular lactate levels only decreased in the AR-C condition in neurons (Fig. 4*E*), confirming that inner retinal neurons take lactate up through MCT2 under basal culture conditions. Conversely, the intracellular lactate levels increased under all conditions in MCs after inhibition of MCTs (Fig. 5, *C* and *D* left). This result suggests that a lactate shuttle from MCs to inner retinal neurons operates, at least under specific conditions (40). Remarkably, when the retina was depolarized, lactate decreased significantly in neurons under all conditions, even when MCT1 (145.5 ± 169.5%, *p* = 0.046) and MCT1/MCT4 (129.9 ± 98.2%, *p* = 0.042) were inhibited. The decrease after inhibition of MCT2 (105.7 ± 90.9%, *p* = 0.039) suggests that depolarization triggered an increase in lactate consumption. In contrast, in MCs, when the retina was depolarized, lactate increase ceased, leading to a new and higher intracellular lactate baseline (Fig. 5, *C* and *D* right, SR *p* = 0.016, AR-C *p* = 0.016, and Syro *p* = 0.016). These results could reflect a flexible and dynamic regulation of glucose and lactate consumption in the inner retina, depending on overall retinal activity status.

## Discussion

Although photoreceptors are the cells with the highest energy consumption in the retina (3, 4), it is equally important to understand the metabolism of inner retinal cells, which face the challenge of being further away from the main choroidal blood supply. MCT2 expression in the inner retina is not surprising because this transporter is known as the neuronal MCT. Several authors have proposed the neuronal consumption of lactate taken up through this specific transporter isoform (21, 41), which is in line with prior immunohistochemical studies in the retina (42, 43). However, a demonstration of the function of this transporter and its role in inner retinal physiology was missing. In vascular retinas, inner retinal cells can obtain metabolites from alternative sources (5), and previous studies have demonstrated the ability of the neuroretina to be metabolically flexible to meet its energy demands (6). Although that study focused on the outer retina, some important enzymes involved in different metabolic pathways were expressed in the INL, supporting a similar metabolic flexibility in the inner retina. Here, we found that under prolonged MCT blockage, RBCs displayed multiple functional alterations, including increased cell death, indicating the dependence of RBCs on extracellular lactate levels. However, since photoreceptors are more sensitive to metabolic disruptions (6), we cannot rule out the possibility that the observed sensitivity of RBCs to a disruption of lactate flux might be secondary to the death of photoreceptors.

Many studies have suggested a dysregulation of calcium and sodium homeostasis under metabolic disturbances (32, 44), because of the strong dependence of ionic gradients across the cell membrane on sustained ATP production (45). Since the transport of ions across biological membranes against concentration gradients is energy-intensive, a disruption in metabolism will affect the restoration of ion gradients (30). Indeed, a delayed return to baseline was observed here in sodium signaling, supporting this idea. This sluggish return to baseline can produce an accumulation of these ions in the cytoplasm, leading to a reduced (and slower) influx of different ions, specifically in calcium signaling (32). An accumulation of intracellular calcium may underlie the depolarization observed after AR-C and mannitol application in RBCs, as seen in electrophysiology experiments, and is likely the reason for the increased cell death observed after prolonged exposure in explant cultures. It is important to note that the alterations observed in the electrophysiological experiments may have been produced by reduced responses of other inner retinal neurons such as ACs, since the reciprocal feedback signal in BCs was diminished (Fig. 3*D*), yet it remains uncertain whether this effect is postsynaptic or presynaptic in origin.

Regarding the substantial increase in sodium responses, a drop in the sodium baseline concentration could explain these results. Many studies have shown that an increase in cytoplasmic calcium concentration directly leads to an increase in mitochondrial calcium levels, which regulates numerous enzymes (46, 47). Subsequently, to recycle calcium ions, mitochondrial Ca^2+^ efflux is mediated by an electrogenic Na^+^/Ca^2+^ exchanger (NCLX) (48, 49), causing a decrease in sodium levels. In addition, this reduction in cytoplasmic sodium may be enhanced following depolarization caused by high intracellular calcium levels, leading to an upregulation of ion pumps that produce a sodium efflux (45). A previous study proposed noncanonical modulation of ATP levels mediated by the Na^+^/K^+^ pump, where the Na^+^ flux could control glycolysis and ATP production (39). Therefore, the regulation of calcium and activation of Na^+^ pumping is consistent with a reduction in cytoplasmic sodium levels.

When ion gradients are affected, alterations in different electrical responses should be expected. Some studies have shown that ERG components are sensitive to glucose deprivation and various metabolic stressors (1, 50). Specifically, inhibition of MCTs with α-cyano-4-hydroxycinnamic acid attenuated the b-wave with delayed implicit response time. However, in the presence of extracellular lactate, a partial recovery was obtained (14). These results suggest a possible consumption of extracellular lactate by ON BCs to maintain electrical responses to visual stimuli. Our results support this notion, as the inhibition of MCT2 caused a reduction in outward and calcium currents and depolarized the membrane potential in RBCs. Additionally, the reduction of the currents and of the membrane potential by glucose deprivation is countered in the presence of extracellular lactate (51). However, our data support the notion that glucose is preferred over lactate to maintain RBC physiological activity (52, 53). We hypothesize that combined lactate and glucose consumption may allow for faster adaptation to changes in neuronal energy demand (54). Since BC activity depends on multiple interactions with other retinal neurons that modulate their cell membrane potential depending on different and rapidly changing light conditions, this could lead them to shift to the faster process of lactate oxidation to pyruvate under certain conditions to sustain their physiological activity (6). Further investigation is necessary to determine whether the lactate consumed is involved in direct ATP production or makes a significant contribution to the tricarboxylic acid cycle.

**Figure 6:**
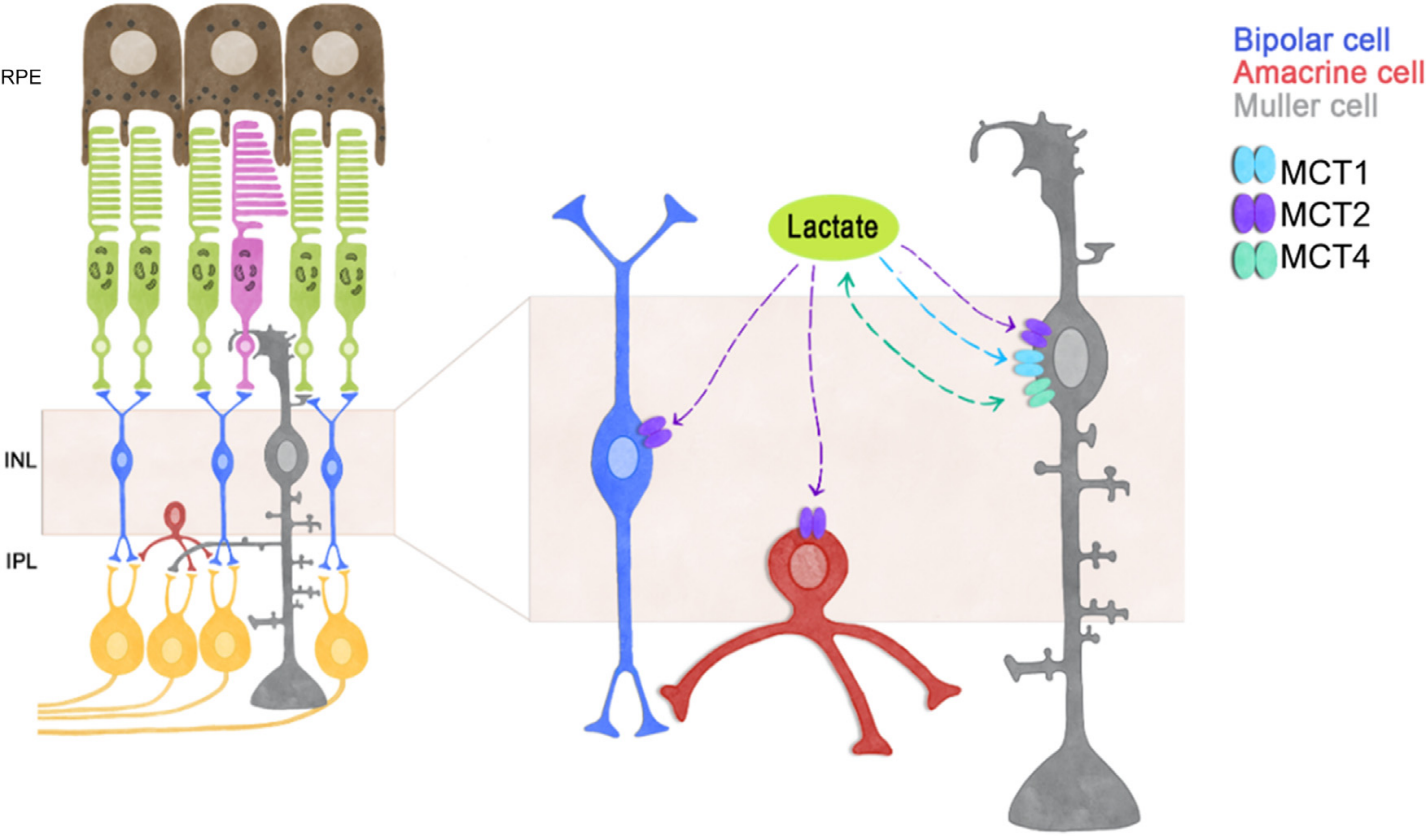
**Model for the lactate dynamics in inner retinal cells.** General model proposing a consumption of lactate by inner retinal cells. Previous work (35) demonstrated the functional expression of MCT1, MCT2, and MCT4 in MCs, where MCT2 (and to a minor degree also MCT1) mainly regulates lactate influx, while MCT4 mediates lactate efflux, contributing to the accumulation of extracellular lactate. Here, we propose that this extracellular lactate produced by MCs and possibly other retinal cell types is consumed by BCs and ACs through MCT2. ACs, amacrine cells; BCs, bipolar cells; MCs, Müller cells; MCT1, monocarboxylate transporter one; MCT2, monocarboxylate transporter 2; MCT4, monocarboxylate transporter 4.

Our results demonstrate lactate consumption by inner retinal neurons which is exacerbated by depolarization. Thus, in addition to the previous results in MCs, this study reveals that a retinal lactate shuttle from Müller glial cells to neurons might operate under our experimental conditions (10) (Fig. 6). However, some loose ends remain. As has recently been reported, photoreceptors also produce lactate (12, 55); therefore, it remains to be defined under which physiological conditions (*e.g.*, light intensities) lactate synthesis in either cell type is induced. In addition, some studies have indicated that experimental conditions might affect the glycolytic rate in MCs, with MCs in primary culture being more glycolytic than MCs in isolated retinas (17, 56). Hence, while lactate production by MCs remains controversial, our results from retinal explant cultures support it. In addition, lactate consumption was also seen in some MCs (Fig. 5, *C* and *D* right) suggesting that some MCs could also be net lactate consumers (11, 12) (Fig. 6).

Interestingly, not all neurons displayed lactate consumption after depolarization (Fig. S6), which reflects the large heterogeneity of BCs and other cells in the INL (57). BCs can be classified according to their response to light as ON-BCs- or OFF-BCs. ON-BCs depolarize upon an increase in light intensity, whereas OFF-BCs depolarize in response to a decrease in light intensity. Because our experiments were performed under photopic conditions, the cells that displayed lactate consumption probably corresponded to ON-BCs (*e.g.*, RBCs). It is important to note that this does not mean that OFF-BCs do not consume lactate, but further experiments will be necessary to determine it.

It is important to emphasize that our study has limitations. For instance, all measurements were obtained from cell bodies, whereas the long cellular projections of MCs, BCs, and ACs might be metabolically isolated and could display different dynamics. Furthermore, the experimental conditions used here for some experiments (retinal explants) might not accurately reflect the physiological conditions of the *in vivo* retina. While retinas were light-adapted, photoreceptors can show variations in their activity after prolonged time in culture depending on the status of the RPE (58, 59). Finally, given that our experiments were carried out under photopic conditions, it is also possible that this metabolic model only applies under this condition.

In summary, the observations reported here suggest that the inner retina participates in a retinal lactate shuttle, where MCs release lactate under organotypic basal conditions, and inner retinal neurons consume a percentage of this extracellular lactate to meet their energy demands. (Fig. 6).

## Experimental procedures

### Animals

WT C57BL/6 mice were housed under standard white cyclic illumination, with water and food *ad libitum*, and were used irrespective of gender. All efforts were made to minimize the number of animals used and their suffering. Protocols complied with the German law on animal protection and were reviewed and approved by the ‘‘Einrichtung für Tierschutz, Tierärztlicher Dienst und Labortierkunde’’ of the University of Tübingen and the bioethics committee of the University of Valparaíso, in accordance with the Chilean animal protection law No. 20.380. To isolate the eyes, animals were deeply anesthetized by inhalation using isoflurane and euthanized *via* decapitation. We used postnatal day 9 (P9) mice to prepare retinal explants, and they were maintained in culture for 7 days, so the retinas were roughly equivalent to P16 on the day of data collection for Figure 1. For Figures 2 and 3, we used acutely isolated retinas at P30. Finally, for the nanosensor experiments, we cultured retinas at P9, and the transfection was made at P11/P12. The experiments were performed after 14 days in culture, between P21 and P28. Thus, all experiments were performed between P16 and P30 (young mice).

### Organotypic retinal explant culture

Retinal explants obtained from P9 WT mice were cultured as previously described (60). Briefly, the retinas were treated for 15 min with 0.12% Proteinase K (Sigma-Aldrich, Cat. No. P2308) at 37 °C for isolation of the retina together with the RPE. Then, the eyes were placed for 5 min in Dulbecco's modified Eagle's medium with 10% fetal bovine serum to deactivate Proteinase K. Finally, the retina was placed with the RPE facing down on cell culture inserts (Millicell, Cat. No. PICM0RG50, Merck Millipore) and supplemented with Dulbecco's modified Eagle's medium (Thermo Fisher Scientific, Cat. No. 31600034) containing 10% fetal bovine serum (Sigma-Aldrich) and 15 mM glucose, which was replaced every 2 days. The cultures were incubated at 37 °C in 5% CO_2_, and 95% humidity for 14 days in a water-jacketed incubator (Thermo Fisher Scientific).

### Immunostainings

Retinas from P30 mice were fixed in 4% paraformaldehyde for 45 min, washed in PBS buffer, cryopreserved with a series of solutions containing 10, 20, and 30% sucrose before being embedded in tissue-freezing medium, and stored frozen at −20 °C. Transverse sections of 14 μm thickness were obtained with a cryostat (Leica CM1900) and deposited on poly-L-lysine-coated slides, which were dried at 37 °C for 30 min and rehydrated for 10 min in PBS at room temperature (RT). For immunofluorescent labeling, the slides were incubated with blocking solution (10% normal goat serum, 1% bovine serum albumin in 0.3% PBS-Triton X 100) for 1 h at RT. The primary antibodies, anti-MCT2 (SLC16A7) (Alomone labs, Cat. No. AMT-012, RRID: AB_2340997), Anti-protein kinase Cα (Thermo Fisher Scientific, Cat. No. MA1-157, RRID: AB_2536865), and anti-calretinin (Abcam, Cat. No. A85366, RRID: AB_2748943) were diluted one:100 in blocking solution and incubated at 4 °C overnight. The slides were washed three times for 10 min each with PBS before the application of the secondary antibody, diluted one:350 in PBS, and were incubated for 1 h at RT. Finally, the slides were washed with PBS and covered in Vectashield with 4′,6-diamidino-2-phenylindole (DAPI) (Vector).

### TUNEL assay

Fixed slides from retinal explant cultures were dried at 37 °C for 30 min and washed in PBS at RT for 15 min. Afterward, the slides were placed in Tris buffer with proteinase K at 37 °C for 5 min to inactivate nucleases. The slides were then washed with Tris buffer (10 mM Tris-HCL, pH 7.4), 3 times for 5 min each. Subsequently, the slides were placed in ethanol–acetic acid mixture (70:30) at −20 °C for 5 min followed by three washes in Tris buffer and incubation in blocking solution (10% normal goat serum, 1% bovine serum albumin, and 1% fish gelatin in 0.1% PBS-Triton X100) for 1 h at RT. Lastly, the slides were placed in the TUNEL solution (labeling with either fluorescein or tetra-methyl-rhodamine; Roche Diagnostics GmbH) in 37 °C for 1 h and covered in Vectashield with DAPI (Vector) thereafter.

### Lactate measurements

Lactate concentration in the culture medium of retinal explants was determined using a commercial blood lactate monitoring system (Edge model S54108, ApexBio). Lactate concentration in the culture medium was measured at days 1, 3, 5, and 7 of culture, prior to medium replacement. Each measurement was taken twice for each explant, and measures are presented as the average.

### Microscopy and cell counting

Fluorescence microscopy was performed with a Z1 Apotome microscope equipped with a Zeiss Axiocam digital camera (Zeiss). Images were captured using Zen software (Zeiss, https://www.zeiss.com/microscopy/en/products/software/zeiss-zen-lite.html) and the Z-stack function (14 bit depth, 2752∗2208 pixels, pixel size = 0.227 μm, 9 Z-planes at 1 μm steps). The raw images were converted into maximum intensity projections using Zen software and saved as TIFF files. Inner retinal cells stained by the TUNEL assay were counted manually on three images per explant, the average cell number in each INL area was estimated based on DAPI staining and used to calculate the percentage of TUNEL positive cells.

### Retinal slice preparation for imaging and electrophysiological experiments

For FRET experiments, the explants were separated from the culture inserts and placed in a chamber with extracellular solution. For imaging and electrophysiology experiments, P30 mice were used. Eyes were enucleated, and the retina was kept in extracellular solution during the remainder of the procedure. The eye was cut along the ora serrata to separate the anterior and posterior chambers, and the retina was separated carefully from the choroid-sclera. The extracellular solution for maintaining both types of slices contained (in mM) the following: 119 NaCl, 23 NaHCO_3_, 1.25 NaH_2_PO_4_, 2.5 KCl, 2.5 CaCl_2_, 1.5 MgSO_4_, 20 glucose, and 2 Na^+^ pyruvate, aerated with 95% O_2_ and 5% CO_2_, pH 7.4. Subsequently, the tissue was embedded in type VII agarose (Cat. No. 39346-81-1 Sigma-Aldrich) dissolved in a solution composed of (in mM): 119 NaCl, 25 Hepes, 1.25 NaH_2_PO_4_, 2.5 KCl, 2.5 CaCl_2_, and 1.5 MgSO_4_ at a pH of 7.4. Finally, the tissue was sliced with a vibratome (Leica VT1000S) to 200 μm thickness. The slices were transferred to the microscope recording chamber, kept in place using a U-shaped platinum wire and superfused with oxygenated extracellular solution at RT (20 °C) under photopic conditions.

### Calcium and sodium imaging

For imaging experiments, 200 μm thick retinal slices were obtained as previously described (23), and incubated for a period of 1 h in a dark room in 2 ml of extracellular solution containing 5 μM fluo-4 AM (Thermo Fisher Scientific) or 7 μM CoroNa Green AM (Thermo Fisher Scientific) in 0.04% pluronic acid. Afterward, the slices were imaged as previously described (23). Relative fluorescence intensities (ΔF/F0) of the ROIs were obtained by dividing all images by the initial (prepulse) image of the series. In this study, all ROIs were chosen from the cell bodies of putative inner retinal neurons, located in the INL. To decrease the effect of photobleaching, periods of light stimulation lasted for 1 s and were followed by 10 s of darkness for the duration of the experiments. For MCT inhibition experiments, the retinal slices were incubated for 15 min with 0.5 μM SR-13800 (Tocris, Cat. No. 5431), 2 μM AR-C155858 (Tocris, Cat. No. 4960), 4 μM Shikonin (Sigma-Aldrich, Cat. No. S7576), or 15 μM FX-11 (MedChem Express, Cat. No. HY-16214) and then stimulated with 12 mM KCl through bath perfusion.

### Electrophysiology

Retinal slices from P30 *ex vivo* retinas were visualized with an upright microscope (Nikon Eclipse FN1) equipped with a 40× water-immersion objective, infrared differential interference contrast, and a digital camera (TCH 1.4 LICE, Tucsen Photonics). Images were captured with ISCapture (https://iscapture.software.informer.com/4.1/) software and processed with Adobe Photoshop CS (Adobe Systems Incorporated). Patch clamp recordings were made from RBCs, whose tentative identity was corroborated by comparing the axon terminal stratification within the inner plexiform layer after dialysis of Lucifer yellow through the patch pipette. Standard intracellular solution contained (in mM) 125 K^+^ gluconate, 10 KCl, 10 Hepes, 2 EGTA, 2 Na_2_ATP, 2 NaGTP, and 1% Lucifer yellow. Recording electrodes were fabricated using borosilicate glass capillaries (1.5 mm OD, 0.84 mm ID; WPI) and pulled to resistances between 10 to 15 MΩ on a Flaming/Brown electrode puller (Sutter P-97). Experiments were only performed if the patch seal resistance was above 1 GΩ and series resistance below 30 MΩ. Signals were amplified with an EPC7 plus patch clamp amplifier (HEKA Elektronik), digitized and sampled at 10 kHz with an A/D board (Digidata 1550, Molecular Devices). Recordings were acquired using the software PClamp 10.4 (Molecular Devices).

For patch clamp experiments, the retinal slices were incubated for 15 min with either SR-13800 or AR-C155858, and then different protocols were applied. Between the experiments, cells were held at a resting membrane voltage of −60 mV. To measure outward currents, 10 mV voltage steps (200 ms duration) from −100 mV to 40 mV were applied to obtain the voltage-dependent current patterns. To measure voltage-activated Ca^2+^ currents, cells were patched with an internal solution containing (in mM) 90 Cs-methanesulfonate, 20 TEA-Cl, 10 Hepes, 10 EGTA, 10 Na_2_-phosphocreatine, 2 MgATP, and 0.2 NaGTP with pH adjusted to 7.4 with CsOH. To obtain the currents, cells were held at a resting membrane voltage of −60 mV and 5 mV voltage steps (100 ms duration) from −70 mV to +20 mV, were applied. Finally, to study the membrane potential, the current was clamped at 0 pA, and the average of 1 min of recording was used to define the membrane potential.

### FRET measurements

At P11 and P12, the explants were transduced by overnight incubation with 5 × 10^6^ plaque-forming units of Ad Laconic, AAV-Laconic, or FLII12Pglu-700Δ6, and imaged after 2 weeks in culture. Adenoviral serotype vectors encoding FRET nanosensor Ad FLII12Pglu-700Δ6 and Ad Laconic were a gift from Dr Ivan Ruminot from the Centro de Estudios Científicos in Valdivia, Chile. The AAV-GFAP-Laconic (Laconic: Addgene #44238; hGFAP promoter fragment: https://doi.org/10.1002/glia.20622) and AAV-hSYN-Laconic (Laconic: Addgene #44238) were constructed by the viral vector facility of ETH Zurich. Since the lactate and glucose sensors used here have similar excitation/emission spectra (Laconic: 460/492 nm for mTFP and 515/526 for Venus; Δ6: 440/480 nm for cyan aequorea fluorescent proteins and 513/530 nm for yellow fluorescent protein), retinal slices were excited at 430 nm and visualized at 480 nm and 530 nm peak wavelength, as previously reported (18). All experiments were performed at RT (22–25 °C) with an upright fluorescence microscope (Olympus BX51) equipped with a 40x water-immersion objective, an Optosplit II emission image splitter, and a Sensicam QE digital camera (Cooke Corp).

FRET experiments were performed in extracellular solution containing (in mM) 119 NaCl, 23 NaHCO_3_, 1.25 NaH2PO_4_, 2.5 KCl, 2.5 CaCl_2_, 1.5 MgSO_4_, 5 glucose,1 lactate, aerated with 95% O_2_ and 5% CO_2_, pH 7.4. The lactate and glucose concentrations were chosen not to saturate the FRET sensors. Data acquisition was performed by custom software written in Python 4.0.1 (https://www.anaconda.com/download#downloads). At the end of the experiments, data were exported for off-line analysis of fluorescence intensities from each channel. To obtain the FRET ratio for Laconic (mTFP/Venus) and Δ6 (yellow fluorescent protein/cyan aequorea fluorescent proteins), fluorescence intensity values from each ROI and background was measured in ImageJ, version 1.52p (NIH, RRID: SCR_003070, https://imagej.net/ij/). For these experiments, all ROIs were chosen from the somas of MCs and putative inner retinal neurons. The FRET data are displayed as the relative FRET ratio, in percentage of change over time of single experiments.

To calculate the delta ratio between the depletion and the saturation for both sensors, the minimum response (depletion) was obtained during the application of 10 mM pyruvate, and the maximum response (saturation) during 10 mM lactate stimulation, their difference yielding the delta ratio. To evaluate the effect of different MCT inhibitors on lactate influx, the positive amplitude peak was measured before and after drug application. The lactate responses were first normalized per cell based on the control response and then averaged throughout the experiments. Finally, the slopes of the responses were calculated by performing a linear regression and obtaining the slope *via* the linear function: f(x)=mx+b, where ***m*** is the slope. Statistical analysis was performed using GraphPad Prism software (RRID:SCR_002798, https://www.graphpad.com/).

### Pharmacology

Since there is only a limited number of commercial MCT inhibitors available, we used three potent and specific inhibitors to isolate MCT isoforms: SR-13800 for MCT1 (SR; Tocris, Cat. No. 5431), AR-C155858 (AR-C; Tocris, Cat. No. 4960) for MCT1 and MCT2, and Syrosingopine (Syro; Sigma-Aldrich, Cat. No. SML1908) to inhibit MCT1 and MCT4. To study intracellular enzymes involved in lactate production, we used Shikonin (Sigma-Aldrich, Cat. No. S7576) to inhibit the M2 isoform of pyruvate kinase, and FX-11 to inhibit LDH A (Medchem Express, Cat. No. HY-16214).

### Data analysis

All data were first analyzed for normality using the Shapiro–Wilk test. If the test determined that the data did not conform to a normal distribution, significant differences were established with the Wilcoxon Matched-Pairs Signed Ranks Test, and Kruskal-Wallis test followed by a Dunn´s multiple comparison test. When the Shapiro-Wilk test determined that the data conformed to a normal distribution, significant differences were established with the paired *t* test and one-way ANOVA, followed by Tukey's multiple comparisons test. The α value was set to 0.05. Unless otherwise stated, data values are given as mean ± standard deviation (SD). Significance levels as indicated by asterisks are: ∗ *p* <0.05; ∗∗*p* <0.01, ∗∗∗ *p* <0.001. Statistical analysis was performed using GraphPad Prism software (RRID:SCR_002798).

## Data availability

All data generated or analyzed during this study are included in this published article (and its Supporting information files). Data reported in this article will be shared by the lead contact upon request.

## Supporting information

This article contains supporting information.

## Conflict of interest

The authors declare that they have no conflicts of interest with the contents of this article.
