# Supplementary material for: Extracellular lactate as an alternative energy source for retinal bipolar cells
Source: J Biol Chem. 2024 Feb 24;300(4):106794. doi: 10.1016/j.jbc.2024.106794 (PMC10966802; doi:10.1016/j.jbc.2024.106794)
Supplement: Supporting Figures S1–S7 [file mmc1.pdf]

Supplementary figures

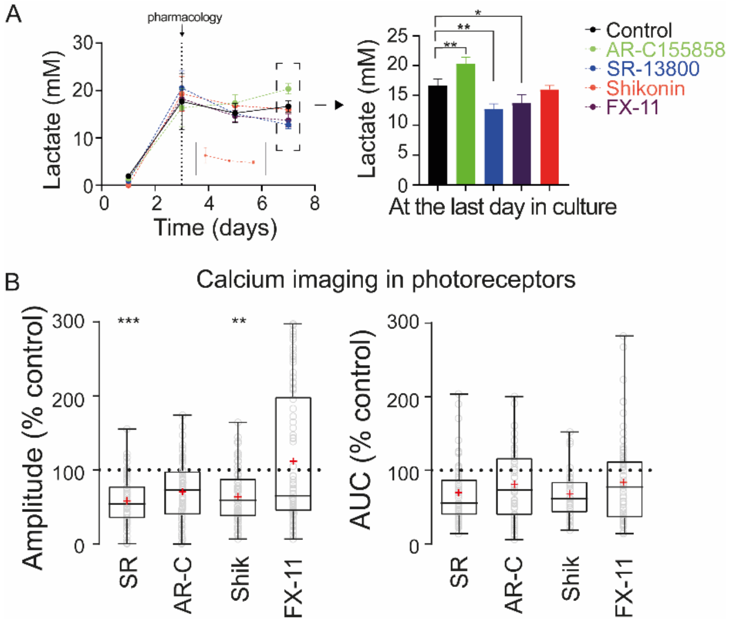

Figure S1. Effect on lactate concentration in retinal explant cultures and on photoreceptor responses in acute retinal slices under inhibition of different enzymes and transporters related to lactate metabolism. (A,Left) Comparison of the lactate concentration in the culture medium of retinal explant cultures at different time-points and under different pharmacological conditions. The vertical dashed line separates between pre-treatment (left) and post treatment with the pharmacologic agents (right). The inset shows the decrease of 3.2 ± 2.1 mM of lactate after 4 days of culture with Shikonin. **(A,Right)** Statistical analysis of lactate concentration at the last day in culture. The control explants treated with DMSO (vehicle) displayed 16.63 ± 1.15 mM lactate. The explants treated with AR-C155858 (MCT2 inhibition) showed an accumulation of lactate, reaching 20.33 ± 1.10 mM ($p = 0.0056$), while the addition of SR-13800 (MCT1 inhibition) and FX-11 (LDH-A inhibition) produced a reduction in the lactate concentration with 12.75 ± 0.83 mM ($p = 0.0041$) and 13.72 ± 1.41 mM ($p = 0.0236$), respectively. The treatment with shikonin does not induce a change compared to control explants (16.02 ± 0.66 mM; $p = 0.8846$), but the cultures treated displayed a reduction in lactate concentration between pre and post treatment (pre-treatment = 19.28 ± 3.62 mM). Graphs show mean ± standard deviation. **(B, left)** Statistical analysis of the amplitude in the calcium imaging experiments in photoreceptors. Significant alterations were observed under SR and shikonin conditions: Under inhibition of MCT1 (SR; $p < 0.0004$) and PKM-2 (Shik; $p = 0.0032$). While no alterations were observed under inhibition of MCT2 (AR-C, $p = 0.0846$), and LDH-A (FX-11, $p > 0.9999$). **(B, right)** Statistical analysis of the area under the curve of calcium responses. No alterations were observed either in SR (69.7 ± 41.3%, $p > 0.9999$), AR-C (81.0 ± 49.0%, $p > 0.9999$), shikonin (68.1 ± 33.4%, $p > 0.9999$) or FX-11 (83.9 ± 56.9%, $p > 0.9999$). Box plots display the median ± min and max values and the mean in red. Individual values are displayed as open circles (gray). The control is presented as a dashed line at 100%, and results are presented as percentage of control. The data were analyzed by Kruskal-Wallis with Dunn's multiple comparison post hoc test. Asterisks indicate * p < .05, **p < .01, ***p< .001. SR = MCT1 inhibitor; AR-C = MCT2 inhibitor; Shikonin = PKM2 inhibitor; FX-11 = LDH-A inhibitor.

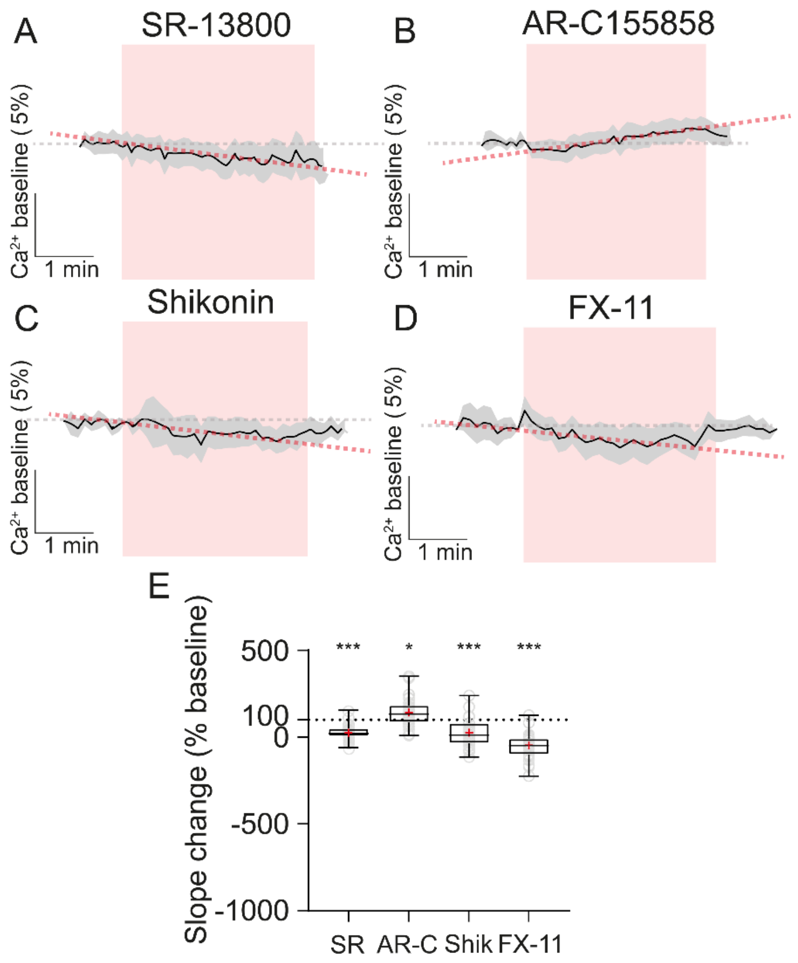

Figure S2. Alterations in relative basal calcium levels during impaired lactate metabolism. (A-D). Traces of an representative experiment in each condition (black line=mean, gray shade=standard deviation, number of cells per experiment: SR = 10 cells, AR-C = 19 cells, shikonin = 15 cells, FX-11 = 22 cells), showing the incubation time with different drugs (red boxes). The gray dashed line shows the slope in the pre-incubation condition, while the red dashed line represents the change in slope during drug incubation. **(E)** Statistical analysis of basal calcium levels. Significant alterations were observed in all conditions: Under inhibition of MCT1 (SR; $p < 0.0001$), PKM-2 (Shik; $p < 0.0001$) and LDH-A (FX-11; $p < 0.0001$), we observed a significant reduction of basal calcium levels. However, inhibition of MCT2 (AR-C; $p = 0.0148$) produced an increase in relative calcium levels. Control is presented as a dashed line at 100%. Box plots show median ± minimum and maximum values and mean in red. Individual values are shown as open circles (gray). Asterisks indicate * $p < .05$, ***$p < .001$. SR = MCT1 inhibitor; AR-C = MCT2 inhibitor; Shikonin = PKM2 inhibitor; FX-11 = LDH-A inhibitor.

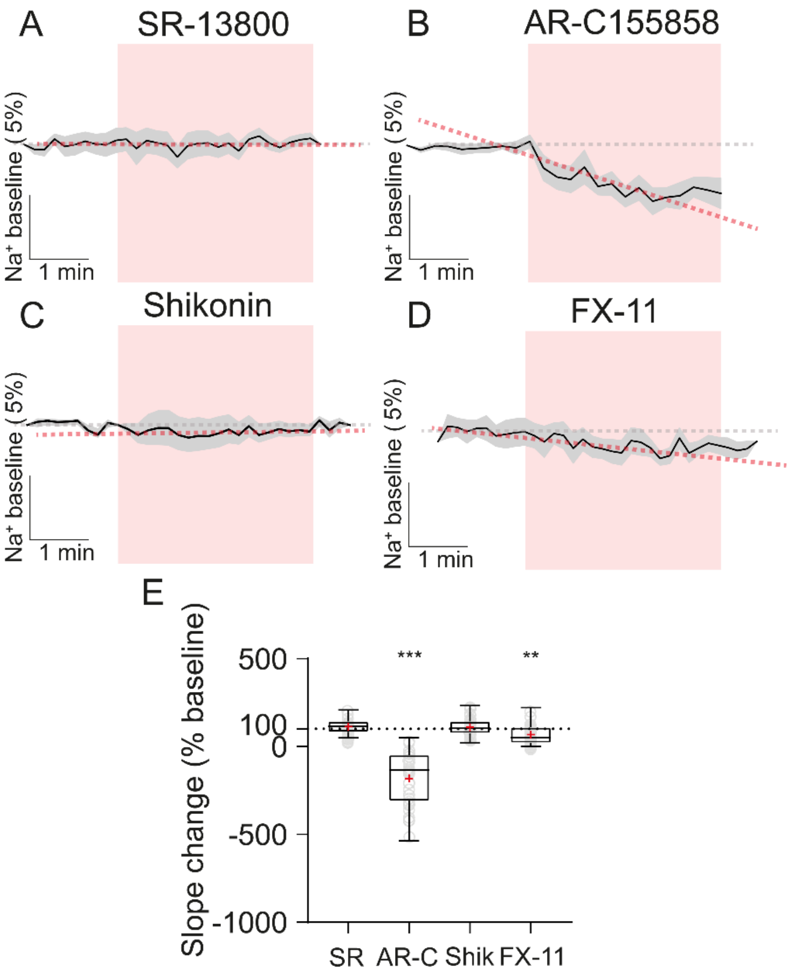

Figure S3. Disruption of lactate metabolism decreased basal sodium levels. (A-D). Traces of an representative experiment in each condition (black line = mean, light gray shadow = standard deviation, number of cells per experiment: SR = 12 cells, AR-C = 13 cells, Shikonin = 12 cells, FX-11 = 17 cells), showing the incubation time with different drugs (red boxes). The gray dashed line shows the slope in the pre-incubation condition, while the red dashed line represents the change in slope during incubation with drugs. **(E)** Statistical analysis of basal sodium levels. Alterations were observed only under two conditions: Under inhibition of MCT2 (AR-C; $P < 0.0001$) and slightly under LDH-A inhibition (FX-11; $P = 0.0014$). While under the blocking of MCT1 (SR; $P = 0.0602$) and PKM2 inhibition (Shik; $P = 0.19$) the basal sodium level was not affected. Control is presented as a dashed line at 100%. Box plots show median ± minimum and maximum values and mean in red. Individual values are shown as open circles (gray). Asterisks indicate * $p < .05$, ** $p < .01$. SR= MCT1 inhibitor; AR-C = MCT2 inhibitor; Shikonin = PKM2 inhibitor; FX-11 = LDH-A inhibitor.

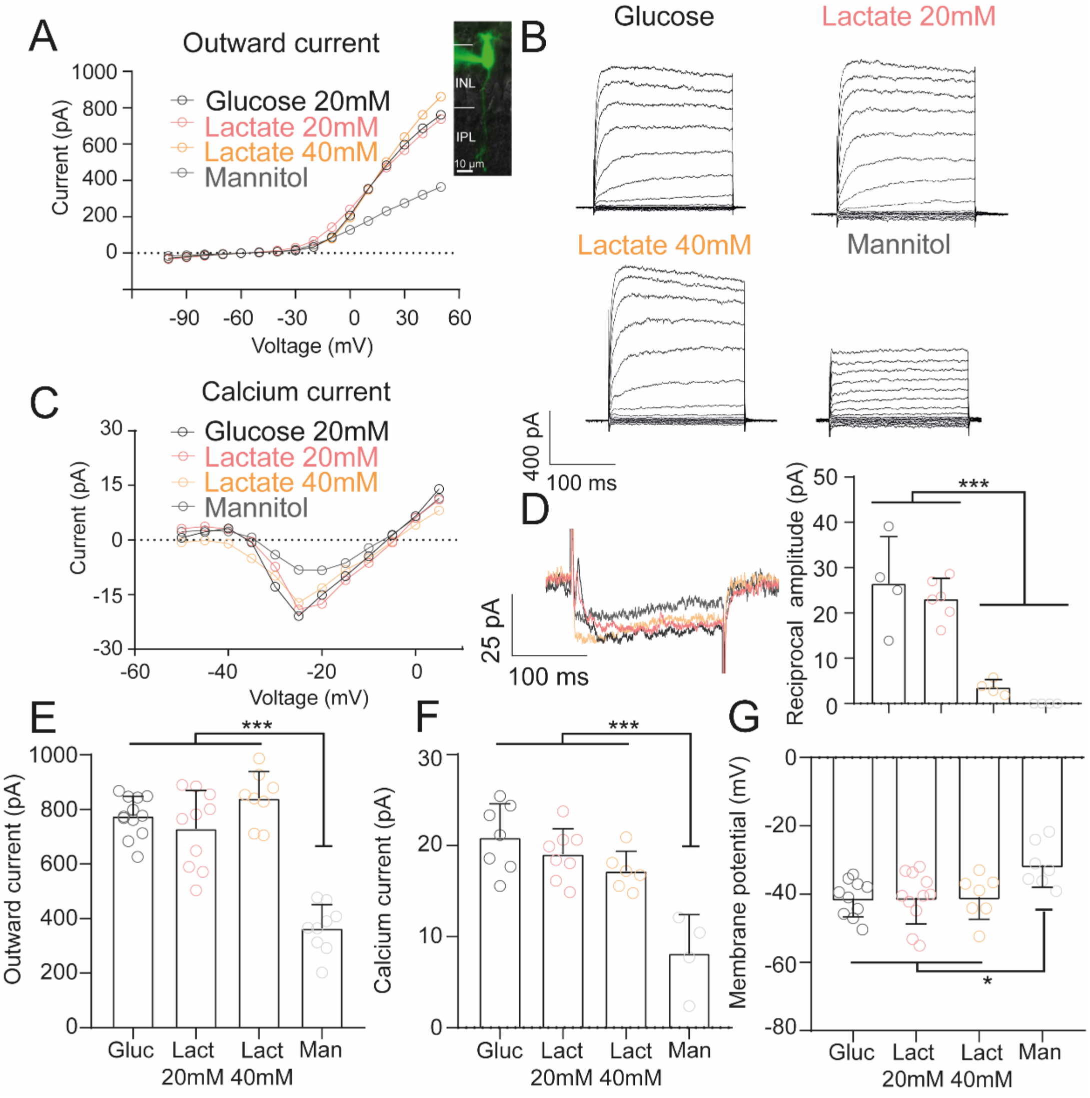

Fig. S4. RBCs can use lactate in the absence of glucose to maintain their current profiles. (A, C), Comparison of the current-voltage relationship of the outward currents and calcium currents under different pharmacological conditions. **(B, D),** Representative responses to depolarizing voltage steps. The reciprocal feedback current was altered only in the mannitol ($p = 0.0002$), and lactate 40 mM conditions ($p < 0.0001$), but was unaffected in lactate 20 mM ($p = 0.7856$). **(E, F)**, In the absence of glucose, a decrease in the outward currents ($p < 0.0001$) and calcium currents ($p < 0.0001$) was observed only in the mannitol condition, but no differences were noted in the lactate 20 mM and 40 mM conditions either in the outward currents ($p = 0.7079$; $p = 0.4973$) or calcium currents ($p = 0.6924$; $p = 0.2019$). **(G)**, Similar results were obtained when we measured the membrane potential, which displayed a depolarization in the mannitol condition ($p = 0.0256$), while it remained unaltered in lactate 20 mM and 40 mM ($p = 0.9999$; $p = 0.9993$). The data were analyzed by one-way ANOVA, with Tukey's multiple comparison post hoc test. Each circle represents a single recorded cell. The data used to plot the outward currents, calcium current, membrane potential and reciprocal feedback for the Lactate 20 mM condition was re-used

from the Fig. 3, since It corresponds to the same condition. Graphs display the mean ± SD; asterisks indicate * $p < .05$, **$p < .01$, ***$p < .001$.

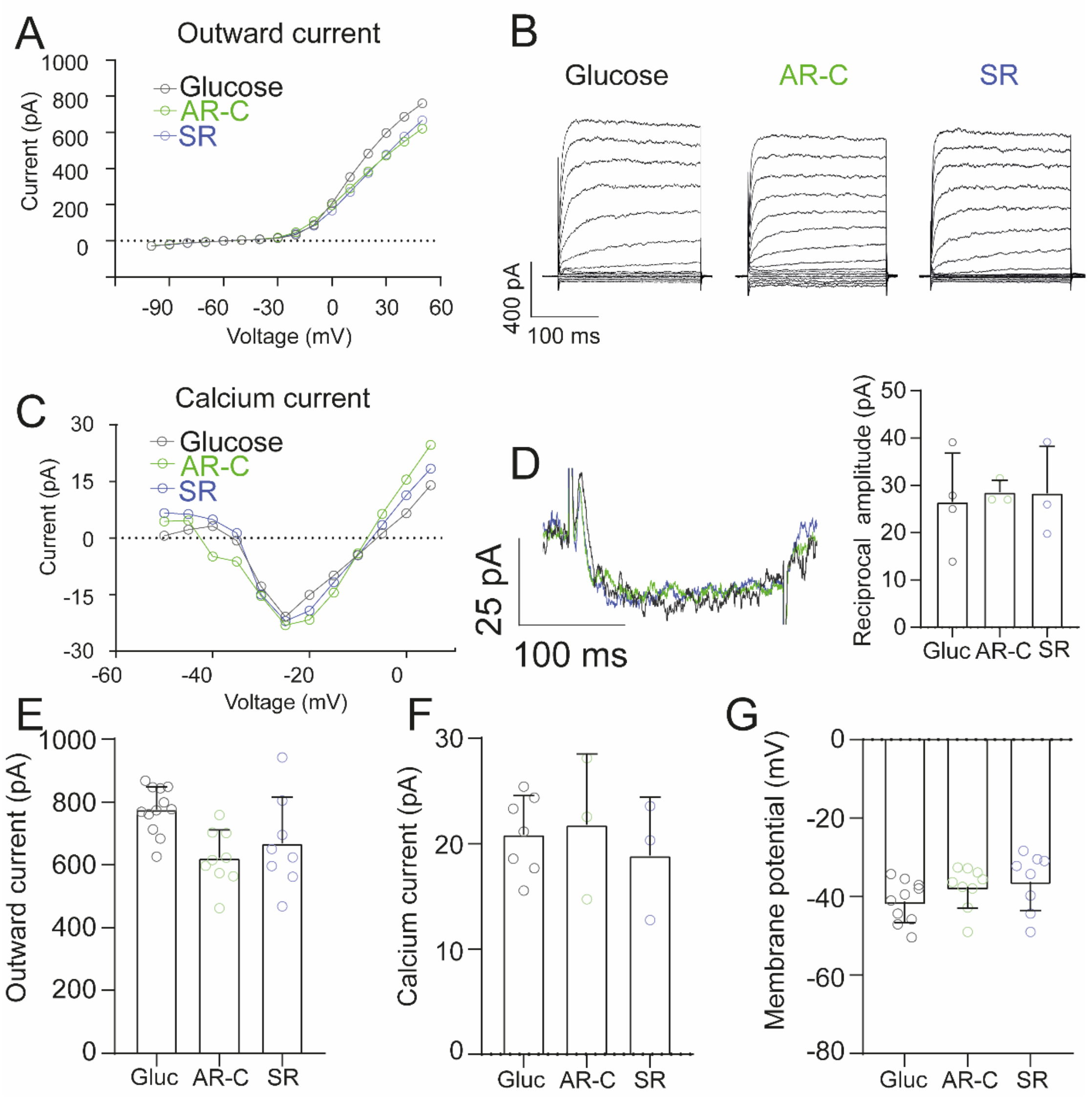

Fig. S5. The inhibition of lactate transport does not affect RBC voltage-gated currents in the presence of glucose. (A, C), Comparison of voltage-current relationship of the outward currents and calcium currents in the different conditions. **(B, D),** Representative responses to depolarizing voltage steps. In the presence of glucose, the reciprocal feedback current was not altered in the AR-C ($p = 0.9501$) and SR conditions ($p = 0.9592$). **(E, F)**, Likewise, neither AR-C ($p = 0.0913$) nor SR ($p = 0.0693$) affected the outward and calcium currents ($p = 0.9581$ and $p = 0.8267$, respectively). **(G)**, Similar results were obtained regarding the membrane potential, with no change caused by either AR-C ($p = 0.3938$) or SR ($p = 0.1921$). The data were analyzed by one-way ANOVA, with Tukey's multiple comparison post hoc test. Each circle reflects a single recorded cell. The data used to plot the outward currents, calcium current, membrane potential and reciprocal feedback for the Glucose condition was re-used from the Fig. S4 since It corresponds to the same condition. Graphs display the mean ± SD. SR = MCT1 inhibitor; AR-C = MCT1 and MCT2 inhibitor.

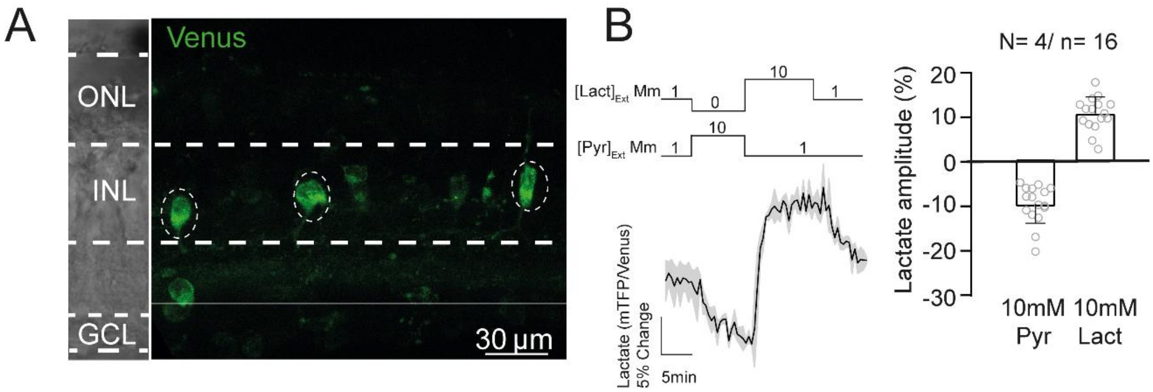

Fig. S6. The Laconic nanosensor can be functionally expressed in inner retinal neurons. (A) Confocal image of Laconic expression in retinal explants after two weeks in culture. Dashed circles show the recorded area. **(B)** Dynamic range of the lactate sensor in inner retinal neurons. ONL= outer nuclear layer; INL = inner nuclear layer; GCL = ganglion cell layer. The black trace represents the average of one experiment (3 cells recorded), while the light gray shadow represents the standard deviation. The number of experiments is presented as: N = number of explants; n = number of cells recorded. Graph displays the mean ± SD.

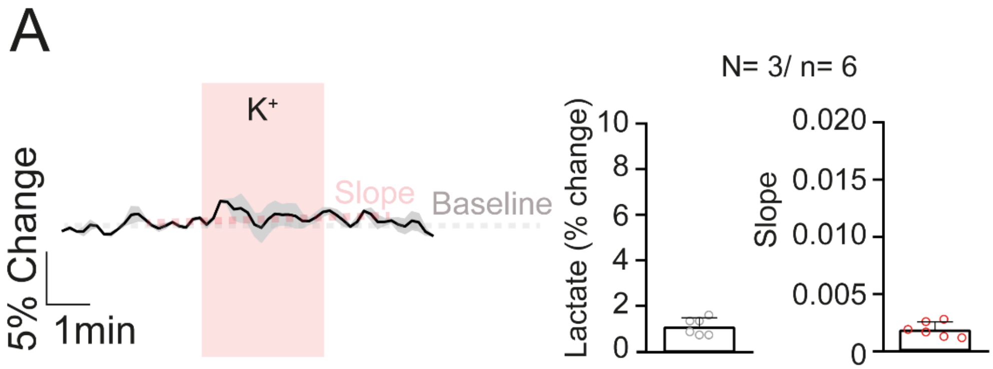

Figure S7. Depolarization by bath application of KCl has no effect on a group of inner retinal cells. (A, left) Representative traces of one experiment showing no responses during and after depolarization. The black trace represents the average of one experiment, while the light gray shadow represents the standard deviation. **(A, right)** Quantification of both amplitude and slope during the bath application of potassium does not demonstrate any alteration of these parameters. Graphs display the mean ± SD. The number of experiments is presented as: N = number of explants; n = number of cells recorded.
